# Practical Evaluation and Management of Insomnia in Parkinson's Disease: A Review

**DOI:** 10.1002/mdc3.12899

**Published:** 2020-02-03

**Authors:** Douglas M. Wallace, William K. Wohlgemuth, Lynn Marie Trotti, Amy W. Amara, Irene A. Malaty, Stewart A. Factor, Sagarika Nallu, Lara Wittine, Robert A. Hauser

**Affiliations:** ^1^ Department of Neurology, Sleep Medicine Division University of Miami Miller School of Medicine Miami FL USA; ^2^ Neurology Service Bruce W. Carter Department of Veterans Affairs Medical Center Miami FL USA; ^3^ Psychology Service Bruce W. Carter Department of Veterans Affairs Medical Center Miami FL USA; ^4^ Department of Neurology and Emory Sleep Center Emory University School of Medicine Atlanta GA USA; ^5^ Department of Neurology University of Alabama at Birmingham School of Medicine Birmingham AL USA; ^6^ Department of Neurology, Fixel Institute University of Florida Gainesville FL USA; ^7^ Jean and Paul Amos Parkinson's Disease and Movement Disorders Center Emory University School of Medicine Atlanta GA USA; ^8^ Department of Pediatrics, Morsani College of Medicine University of South Florida Tampa FL USA; ^9^ Department of Medicine, Morsani College of Medicine University of South Florida Tampa FL USA; ^10^ Department of Neurology, Morsani College of Medicine University of South Florida Tampa FL USA

**Keywords:** insomnia, Parkinson's disease, sleep disturbance, nonmotor symptoms

## Abstract

**Background:**

Insomnia is one of the most common nonmotor features of Parkinson's disease (PD). However, there are few practical guidelines for providers on how to best evaluate and treat this problem.

**Methods and Findings:**

This review was developed to provide clinicians with a pragmatic approach to assessing and managing insomnia in PD. Recommendations were based on literature review and expert opinion. We addressed the following topics in this review: prevalence of insomnia in PD, sleep–wake mechanisms, theoretical models of insomnia, risk factors, assessment, pharmacologic and nonpharmacologic treatments. Insomnia treatment choices may be guided by PD severity, comorbidities, and patient preference. However, there is limited evidence supporting pharmacotherapy and nonpharmacologic treatments of insomnia in PD.

**Conclusions:**

We provide a pragmatic algorithm for evaluating and treating insomnia in PD based on the literature and our clinical experience. We propose personalized insomnia treatment approaches based on age and other issues. Gaps in the existing literature and future directions in the treatment of insomnia in PD are also highlighted.

Insomnia symptoms are among the most common nonmotor symptoms in Parkinson's disease (PD) and are key determinants of quality of life.^1^, ^2^ Importantly, insomnia symptoms have been reported in up to 80% of individuals with PD.^3^, ^4^ To our knowledge, there are no reviews exclusively focused on the assessment and treatment of insomnia in PD. The aims of this review are to provide (1) a comprehensive understanding of insomnia symptoms in PD and (2) guidance concerning its assessment and treatment for health providers caring for individuals with PD.

## Methods

### Search Strategy

Details regarding the search strategy are provided in the Supporting Information Materials and Supporting Information Figure S1.

## Definitions of Insomnia

### Insomnia

According to the *International Classification of Sleep Disorders* 3rd edition criteria, insomnia disorder is defined as complaints of difficulty initiating sleep (DIS), and/or difficulty maintaining sleep (DMS), and/or early morning awakenings (EMA).^5^ These nocturnal symptoms must be accompanied by daytime impairment related to sleeping difficulties. Diagnosis of chronic insomnia disorder requires that the complaints be present at least 3 times per week for at least 3 months. Studies assessing insomnia symptoms in PD have used 1 of the following 2 insomnia definitions: (1) nocturnal complaints alone (DIS, DMS, EMA) or (2) insomnia disorder congruent with *International Classification of Sleep Disorders* 3rd edition criteria. The latter insomnia definition is more restrictive and may lead to lower estimates of insomnia prevalence.^5^ In addition, the broader construct of “poor sleep quality” is sometimes equated to insomnia, although this may capture disrupted sleep regardless of etiology.

## Assessment of Insomnia

### Clinical Interview

A clinical interview is the gold standard for establishing a diagnosis of insomnia disorder. The interview gathers information on the type of insomnia complaint, etiology, duration, and repercussions. It also excludes other potential etiologies of the sleep complaint (eg, medications, environmental disruptors, comorbid sleep disorders). A clinical interview guide for insomnia assessment is provided in Table 1.

**Table 1:** *Clinical interview for insomnia in Parkinson's disease*

Assessment
**Sleep schedule** What time do you usually get into bed?
What time do you usually try to fall asleep?
What time do you usually wake up?
What time do you get out of bed?
Are these times consistent each day?
How many hours of sleep do you get each day?
Would you prefer your bedtime/waketime at later or earlier times than those listed above? If so, when would you prefer?
**Sleep Habits/Hygiene**
Do you take any sleep aids?
If so, how many times each week do you use a sleep aid?
Do you take naps?
If so, how many times each week and how long do your naps usually last?
Do you drink caffeinated beverages every day?
If so, how many times each day and when is your last cup?
Do you drink alcoholic beverages at night?
If so, how many drinks do you consume?
Do you smoke cigarettes or other‐containing nicotine products in the evening?
Is your bedroom environment dark and quiet?

### Insomnia Assessment Instruments

In addition to the interview, psychometrically validated scales can be used. One brief, commonly used scale is the Insomnia Severity Index (ISI), which consists of 7 items with higher scores indicating more difficulty with sleep. This scale retrospectively assesses insomnia during the previous 2 weeks.^6^ The first 3 items assess DIS, DMS, and EMA, respectively. The last 4 items assess the daytime repercussions of the nocturnal complaints. In community‐based general population samples, an ISI score ≥ 10 has a sensitivity of 86.1% and a specificity of 87.7 % for detecting insomnia disorder.^7^

In contrast to the ISI, which assesses sleep retrospectively, a sleep diary provides a prospective method to track an individual's sleep pattern over time. Individuals are asked to record their bed/rise times, total sleep time, sleep onset latency, number of awakenings, and sleep quality every morning.^8^ Other sleep behaviors (napping, irregular wake‐up times) can be extracted from reviewing these data. A limitation of this method is that completion by individuals with cognitive impairment can be difficult; however, caregivers may be able to complete the diary. Several validated paper‐and‐pencil or electronic sleep diaries are available free of charge.^8^

Sleep assessment instruments not exclusive to insomnia include the Pittsburgh Sleep Quality Index (PSQI), Scales for Outcomes in Parkinson's Disease‐Sleep (SCOPA‐S), and the Parkinson's Disease Sleep Scale (PDSS; Supporting Information Materials S1).^9^, ^10^, ^11^, ^12^, ^13^

### Objective Sleep Measures

Although overnight polysomnography (PSG) is not indicated in the routine assessment of adults with insomnia, it may be necessary when comorbid sleep disorders are suspected (eg, sleep disordered breathing [SDB]).^14^ PSG can determine whether SDB, rapid eye movement sleep behavior disorder (RBD), or other sleep‐related movements are contributing to disturbed sleep.

Comparisons of various insomnia assessment methods are provided in the Supporting Information Table S1.

## Prevalence of Insomnia in PD

Epidemiological estimates of insomnia prevalence in the general population are quite variable (6%–30%).^15^ This has primarily been the result of the definition of insomnia being used with more restrictive definitions, leading to lower estimates. Similarly, in PD, reported insomnia rates vary widely from 25% to 80%, partially because of the differences in insomnia definitions and sample characteristics (eg, population based vs. clinic based, PD severity).^16^, ^17^, ^18^, ^19^, ^20^ We present data of insomnia in PD based on the definition of insomnia specific to each protocol.

### Insomnia Complaints in PD

Studies examining insomnia prevalence in PD have found that as PD severity worsens, the frequency of insomnia complaints increases. For example, in a study using the PDSS in individuals with mild PD, Svensson and colleagues^21^ found that 25% of the cohort reported either sleep onset and/or maintenance insomnia. In moderate PD, Tandberg and colleagues^22^ found that individuals with PD had a higher prevalence of DMS (39% vs. 12%, *P* < 0.01), but not DIS (32% vs. 22%, *P* > 0.05), than non‐PD sleepers. In the Parkinson's and non‐motor symptoms (PRIAMO) multicenter study, Barone and colleagues^23^ found that in patients with moderate PD, 36.9% reported insomnia symptoms using a single question on the Non‐Motor Symptom (NMS) questionnaire. Thus, among individuals with PD, DMS appears to be the most common complaint and prevalence increases with worsening PD severity.

### Longitudinal Analyses of Insomnia Complaints in PD

A total of 3 longitudinal studies have documented the fluctuating nature of insomnia symptoms in PD. In a population‐based study, Tholfsen and colleagues^16^ compared insomnia complaints between a newly diagnosed, drug‐naïve PD cohort and controls at 3 time points for 5 years. Insomnia was identified by nocturnal complaints or use of sleeping medications. During the study period, the overall prevalence of insomnia complaints was similar between individuals with PD and controls. Although the prevalence of DMS was equivalent at baseline (34% vs. 32%), follow‐up during 5 years showed an increasing prevalence of DMS for individuals with PD (50%), but not for controls (38%). In another study examining insomnia complaints for 8 years in moderate PD, Gjerstad and colleagues^19^ observed that insomnia was present in 54% to 60% of individuals at each of 3 time points. The prevalence of DIS, DMS, and EMA ranged from 23% to 30%, 23% to 44%, and 19% to 24%, respectively. Of the study participants, 20% developed insomnia. Similarly, in a hospital‐based study following individuals with moderate PD for 5 years, Zhu and colleagues^17^ tracked insomnia complaints at annual assessments. Of the study participants, 27% reported insomnia at baseline, and 51% reported insomnia during at least one time point. These data highlight that insomnia rates in PD change over time and require periodic assessments.

### Insomnia Disorder in PD

Even when stricter diagnostic criteria (nocturnal and daytime symptoms) are applied, insomnia prevalence rates remain elevated in PD. For example, in a large Finnish cross‐sectional study of randomly selected individuals with moderate PD, 43% of individuals fulfilled the chronic insomnia disorder criteria.^18^ Specifically, 18% of individuals reported DIS, 31% reported DMS, 40% reported EMA, and 39% reported nonrestorative sleep.

## Normal Sleep–Wake Mechanisms and Aberrations in PD

Consolidated sleep depends on the coordination of multiple neurophysiological and environmental cues. A theoretical model underlying the cycle of sleep–wakefulness is known as the 2 process model of sleep: (1) process S (homeostatic sleep drive) and (2) process C (circadian influence).^24^ In the morning, homeostatic sleep drive is low but increases with prolonged wakefulness. Acting as a counterbalance to homeostatic sleep drive, circadian drive for wakefulness increases during the morning, peaks during midday, and decreases in the evening. At night, sleep drive peaks and circadian arousal is withdrawn, leading to sleep onset. During sleep, homeostatic sleep drive diminishes and the circadian arousal rhythm begins to increase, which facilitates morning awakening. Conditions that disrupt/upset this balance may result in insomnia symptoms.

A comprehensive review of neuroanatomical structures underlying sleep and wakefulness is beyond the scope of this review; however, we focus here on known degenerative changes in PD impacting wakefulness and sleep consolidation.^25^ Widespread neuronal loss with neurotransmitter deficits across dopaminergic and nondopaminergic systems occur in PD. First, there is the loss of the dopaminergic neurons of the substantia nigra pars compacta, a component of the ascending reticular activating system. Normally, wakefulness is maintained through activation of the cerebral forebrain via the ascending reticular activating system and other wake‐promoting pathways from the posterior hypothalamus, midline intralaminar thalamus, and nucleus basalis. Dysfunction in any of these pathways results in daytime sleepiness with inadvertent episodes of napping, decreasing homeostatic sleep drive at night. Other key structures are also compromised in PD (ventral tegmental area, dorsal raphe nucleus, locus ceruleus, pedunculus pontine nucleus, and lateral hypothalamus), resulting in the loss of coordination of sleep stage transitions and greater sleep instability in PD.^25^, ^26^

Dysfunction of the circadian system in PD, as a consequence of both neurodegeneration and medications, may also contribute to insomnia complaints.^26^ In studies of individuals with early PD, the function of the suprachiasmatic nucleus (SCN) is unchanged, with untreated individuals having similar circadian melatonin profiles to age‐matched healthy controls.^27^ However, among individuals with PD treated with dopaminergic therapy, a number of circadian abnormalities have been described, including a blunted 24‐hour melatonin secretion and a lower amplitude of the melatonin rhythm than controls.^27^, ^28^ These data suggest that some observed abnormalities in melatonin secretion may relate to dopamine replacement therapy itself. In more advanced PD, behavioral factors such as a sedentary lifestyle with limited sunlight exposure may also weaken the circadian system.^26^ Furthermore, anatomical abnormalities in PD, such as optic neuropathy and loss of retinal ganglion cells, may weaken photic entrainment.^29^, ^30^

## Models of Insomnia

There are several models of insomnia: behavioral, cognitive, physiological hyperarousal, neurocognitive, and neurobiological.^24^ Each of these focus on different potential underlying etiologies. One model that helps explain the developmental trajectory of insomnia was proposed by Spielman and is referred to as the 3‐P model (predisposing, precipitating, perpetuating).^24^ Individuals may be predisposed to develop insomnia based on behavioral, cognitive, or biological vulnerabilities. Among vulnerable individuals, a precipitating event (eg, PD onset, major loss) may trigger insomnia. In response to this new sleep problem, individuals develop maladaptive beliefs about sleep (eg, “I am worried that I may lose control over my abilities to sleep”) and counterproductive behavioral strategies (eg, napping, spending excessive amounts of time in bed awake) that can undermine normal homeostatic sleep drive and circadian functioning. Undermining normal sleep functioning will then perpetuate the insomnia.

## Unique Risk Factors for Insomnia in PD

Many of the same demographic risk factors for insomnia in the general population also apply to individuals with PD. For example, women with PD report a greater prevalence of insomnia symptoms than men with PD.^17^, ^18^, ^22^ However, there are also features specific to PD that increase insomnia risk.

### Nocturnal Motor Symptoms

As PD progresses, several of its core motor features can disrupt sleep. Nocturnal bradykinesia and rigidity may cause difficulty turning or repositioning in bed and present challenges to sound sleep.^31^, ^32^ Although resting tremors may improve during sleep, exacerbation of tremor with nocturnal awakenings may contribute to difficulty returning to sleep. Furthermore, nocturnal dystonia can disrupt sleep even in untreated individuals with early PD.^20^ In the study by Zhu and colleagues,^17^ individuals with greater baseline motor fluctuations reported greater insomnia (DIS, DMS, and EMA) over time. Thus, there are several motor phenomena which may impair nighttime sleep and may be addressed with better nighttime management of PD motor symptoms.

### Nonmotor Medical Symptoms

Nocturia and nocturnal pain are 2 common nonmotor conditions in PD linked to insomnia.^23^, ^33^, ^34^ For example, Zhu and colleagues^17^ reported that nocturia was significantly associated with DMS and inadequate sleep time. In another study exploring the role of pain and its relationship to sleep disturbance in PD, pain ratings were positively associated with the Parkinson's disease sleep scale‐2 (PDSS‐2) score.^35^

### Nonmotor Psychiatric Factors

Although not unique to PD, the most consistent finding in studies of neuropsychiatric factors is the association of depression and insomnia.^17,19,33,35^ In adjusted models controlling for PD severity, duration, sex, medications, and other covariates, Gjerstad and colleagues^19^ reported that depressive symptoms were significantly associated with insomnia symptoms. Although anxiety disorders often coexist with depression in PD,^36^ studies controlling for anxiety and depressive symptoms concurrently show that depression may be the stronger predictor of insomnia symptoms in PD.^33^, ^35^ PD‐related psychosis and nocturnal hallucinations can also contribute to sleep disruption.

### Medication Effects

Dopaminergic medications are well known to cause daytime somnolence and sudden episodes of sleep, with higher dosages of dopamine agonists and total levodopa equivalents increasing risk.^37^ However, dopaminergic medications are also associated with insomnia. In rodents, a dose‐dependent effect was demonstrated whereby higher doses of dopamine agonists such as pramipexole induce increased wakefulness during the sleep period, whereas lower doses reduced wakefulness and increased sleep.^38^ Doufas and colleagues^39^ conducted a meta‐analysis of randomized controlled trials (RCTs; for any indication) in which any drug had been evaluated versus placebo and sleep had been assessed and evaluated for insomnia‐related outcomes. They identified 19 medications associated with insomnia. These included the dopamine precursor levodopa; the dopamine agonists ropinirole, rotigotine, pramipexole, and lisuride; and the catechol‐O‐methyltransferase inhibitor tolcapone. Also associated with insomnia were the acetylcholinesterase inhibitors galantamine, donepezil, and rivastigmine; the selective serotonin reuptake inhibitors fluvoxamine, sertraline, and fluoxetine; the serotonin–norepinephrine reuptake inhibitor venlafaxine; and 6 others. Increased but nonsignificant odds ratios were observed for cabergoline, pergolide, rasagiline, entacapone, and amantadine. Thus, both dopaminergic medications and other medications commonly used in PD (antidepressants, cognitive enhancers) may increase the risk of insomnia. However, an important limitation of this meta‐analysis is that not all the data come from trials of PD patients, and it is possible that PD patients may exhibit a different response. In addition to those previously mentioned, other activating medications commonly used in PD (ie, monoamine oxidase inhibitors, modafinil) may contribute to insomnia.^3^, ^4^ Thus, a close examination of the types and dosing schedule of all medications is a key component of evaluating potentially reversible causes of insomnia in PD.

### Behavioral/Psychological Factors

There are limited data examining the relationship of sleep behaviors and beliefs in insomnia in PD. One study showed that individuals with PD took significantly longer daily naps than age‐matched controls.^40^ This excessive napping may significantly weaken the nocturnal homeostatic sleep drive. Another study specifically assessed dysfunctional sleep beliefs in PD and their relationships to sleep quality (PSQI) and objectively measured sleep.^41^ Greater dysfunctional beliefs and attitudes in the domain of worry/helplessness were associated with poor perceived sleep quality. Similar to others with insomnia, excessive worry about losing control of sleep and ruminating about sleep's negative consequences perpetuate the disorder in PD.^24^, ^41^ These few data suggest that behavioral factors are prime targets for behavioral treatment in individuals with insomnia and PD.

### Comorbid Sleep–Wake Disorders

**Figure 1:**
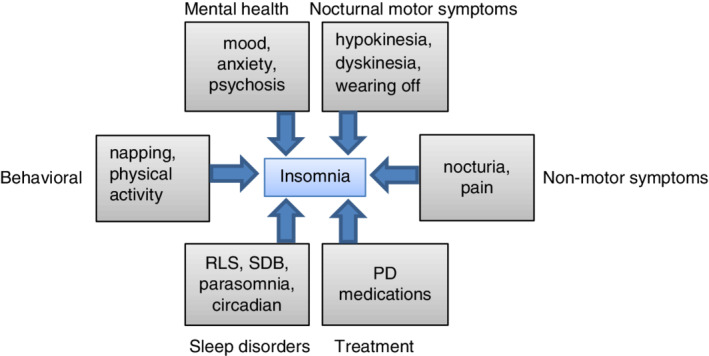
Insomnia risk factors in Parkinson's disease. PD, Parkinson's disease; RLS, restless legs syndrome; SDB, sleep disordered breathing.

Although early studies suggested the SDB may be more common in PD, a meta‐analysis of controlled studies in PD showed that the prevalence of SDB was similar to that of age‐matched controls.^42^ As the estimated prevalence of moderate to severe SDB in those older than age 60 is elevated (men, 24%–65%; women, 16%–35%), all individuals with PD and DMS should be screened for SDB.^43^ In addition, restless legs syndrome (RLS) may be more common in those with PD, with the majority of studies finding increased rates among those with PD (3%–52%) relative to controls (0%–10%).^44^ The uncomfortable sensations of RLS that occur during periods of immobility often prevent sleep, and because they are typically improved with voluntary movement, commonly result in the individual getting out of bed to walk about. Furthermore, up to 90% of individuals with RLS also experience periodic limb movements of sleep, rhythmic limb jerks, which can disrupt sleep continuity.^5^, ^44^ Another common sleep disturbance in PD is RBD, a parasomnia wherein individuals appear to “act out their dreams,” often manifesting violent movements (eg, punching, kicking) and vocalizations associated with vivid dreams.^5^, ^45^ A confirmed diagnosis of RBD requires PSG demonstrating pathologically preserved muscle tone during rapid eye movement sleep (rapid eye movement without atonia).^5^ Although RBD episodes themselves do not typically cause sleep disruption, sleep‐related injuries and/or frightened bed partners can precipitate awakenings and potentially contribute to sleep resistance. In the analysis of Ylikoski and colleagues,^18^ RLS was associated with increased odds of DIS (odds ratio 2.6; 95% confidence interval, 1.4–5.2) while symptoms suggestive of RBD were associated with increased likelihood of disturbed sleep (odds ratio 2.7; 95% confidence interval, 1.4–5.0). Thus, the existing evidence points to RLS/periodic limb movements of sleep and potentially RBD as being comorbid sleep disorder risk factors for insomnia in PD (Fig. 1).

## Pharmacological Treatments of Insomnia in PD

### Dopaminergic Medications for Insomnia in PD

There is limited evidence on the use dopaminergic medications to improve insomnia in people with PD (Table 2). For example, in a group of 40 PD patients with motor fluctuations, the addition of carbidopa–levodopa controlled release (CR) at bedtime was no better than placebo at improving subjective sleep measures, although nocturnal akinesia was improved.^46^ In a polysomnographic study, patients taking carbidopa–levodopa CR at bedtime were also not found to have improved objective sleep parameters (total sleep time, number of awakenings, or sleep stage architecture) when compared with individuals sleeping without carbidopa–levodopa CR (in the *off* state).^47^ Thus, there is little evidence to support adding carbidopa–levodopa CR to specifically address insomnia.

**Table 2:** *Clinical trials examining pharmacologic interventions for insomnia in PD*

Study	Design	Demographic + PD Staging	Intervention	Treatment Duration	Insomnia/Sleep Assessments	Main Findings
Rios Romenets et al., 2013^57^	Randomized clinical trial	N = 18, 78% men; age 66 ± 12 years; H&Y: 1–3	CBT‐I + daily BLT (n = 6) vs. doxepin 10 mg (n = 6) vs. placebo (PLB; red light; n = 6)	6 weeks	PDSS, PSQI, ISI, SCOPA‐S, sleep diary: B, 6 weeks; PGI‐C, CGI‐C, FSS, ESS	Doxepin improved sleep vs. PLB for: ISI (−9 vs. −2*), SCOPA‐night (−5.2 vs. −2.3*), PGI‐C (1.7 vs. 0.5*), CGI‐C (1.4 vs. 0.3**), FSS (−17.0 vs. 0*)
Stocchi et al, 1998^46^	Double‐blind, crossover clinical trial	N = 40; age 66 ± 20 years, H&Y: 2–4; all participants had motor fluctuations	CDLD CR at bedtime (n = 40) vs. PLB	2 weeks, separated by 10‐day washout	Subjective TST, SOL, awakenings, overall sleep	Sleep measures were not significantly improved; only nocturnal akinesia significantly improved with CDLD vs. PLB
Pahwa et al, 2007^48^	Randomized, double‐blind clinical trial	N = 393; 63% men; age 57 ± 11 years; H&Y: 2.7 ± 0.5	Ropinirole PR (2 mg starting dose, up to 24 mg, n = 202) vs. PLB (n = 191)	24 weeks of PR ropirinole vs. PLB	PDSS	Adjusted treatment difference of 4.7 (95% CI 0.8–8.6*) in PDSS favoring ropinirole PR vs. PLB
Chaudhuri et al, 2012^49^	Randomized, double‐blind clinical trial (secondary analysis)	N = 182 for PDSS <100; 60% men; age 66 ± 10 years; H&Y: 2–4	Ropinirole PR (2 mg starting dose, up to 24 mg, n = 93) vs. PLB (n = 89)	24 weeks of PR ropirinole vs. PLB	PDSS, PDSS subscales	Δ in PDSS subscales mean treatment difference global sleep quality 3.0 95% CI 0.6–5.5* and nocturnal motor symptoms 1.7 95% CI 0.5–2.9**
Xiang et al, 2018^50^	Randomized, double‐blind clinical trial (secondary analysis)	N = 119 for PDSS <90; 50% men; age 61 ± 10 years; H&Y: 2–5	Pramipexole (PPX) IR (n = 60) vs. PPX SR (n = 59)	7‐week titration then 11 weeks of PPX IR or PPX SR stable dose	PDSS, PDSS subscales, ESS	Δ in PDSS subscales Total: SR 28.5*** IR 21.7** Nocturnal motor symptoms: SR 9.6** IR: 8.9** Global sleep quality: SR 6.6** IR: 4.3* No differences in Δ of the PDSS or its subscales in SR vs. IR
Poewe et al, 2007^51^	Randomized, double blind, clinical trial	N = 505; 63% men; age 64 ± 10 years; H&Y NR	ROT (2–16 mg; n = 204) vs. PPX (up to 4.5 mg daily; n = 201) vs. PLB (n = 101)	7‐week titration then 16 week stable dosing	PDSS	Improvement in PDSS with ROT vs. PLB (4.3 vs. −2.8**) and PPX vs. PLB (4.9 vs. −2.8***)
Trenkwalder et al, 2011^53^	Randomized, double‐blind clinical trial	N = 287; 64% men; age 64 ± 10 years; H&Y: 1–5; all participants had early morning motor symptoms	ROT (2 mg starting dose, up to 16 mg, n = 191) vs. PLB (n = 96)	8‐week titration followed by 4‐week stable dosing	PDSS‐2, individual PDSS‐2 items and subscales, nocturia, NMSS	Greater improvement in PDSS with ROT vs. PLB (LS mean difference − 4.3***), with improvement in 10/15 PDSS items; greater improvement in NMSS with ROT vs. PLB (LS mean difference − 6.7**), including sleep/fatigue (LS mean difference − 2.0**)
Mizuno et al, 2014^52^	Randomized, double blind, parallel group, clinical trial	N = 414; 41% men; age 66 ± 8 years; H&Y: 2.8 ± 0.6	ROT (2–16 mg; n = 164) vs. ropinirole (up to 15 mg; n = 166) vs. PLB (n = 84)	12‐week titration followed by 4‐week stable dosing	PDSS‐2	Improvement in PDSS‐2 with ROT vs. PLB (LS mean difference ‐2.6***) but equivalent between ROT and ropirinole (−0.7; *P* = 0.28)
Martinez‐Martin et al, 2015^54^	Prospective, observational study	APO group: N = 43; 49% men; age 62 ± 11 years; H&Y 3 IJLI group: N = 44; 57% men; age 63 ± 9 years; H&Y: 4	APO infusion or IJLI	6 months	NMSS, NMSS domains	Improvements in NMSS total score and sleep/fatigue scale for both treatments Relative change (%) in sleep/fatigue domain was greater for IJLI than APO (−48 vs. −24*)
Dowling et al, 2005^58^	Double‐blind, crossover clinical trial	N = 40; 73% men; 62 ± 8 years; H&Y: 1.5–5	Melatonin 5 mg (n = 40) vs. melatonin 50 mg (n = 40) vs. PLB (n = 40)	2‐week treatment, separated by 1‐week washout	ESS, SSS, GSDS, PSQI	TST increased with 50 mg melatonin vs. PLB (10 minutes*); GSDS total scores improved with 5 mg melatonin vs. PLB*
Medeiros et al, 2007^59^	Randomized, clinical trial	N = 18; 78% men; 61 ± 7 years; H&Y: 1–3	Melatonin 3 mg (n = 8) vs. PLB (n = 10)	4 weeks	PSG, PSQI, ESS	PSQI scores significant lower with melatonin vs. PLB (4.5 vs. 8.7*)
Menza et al, 2010^56^	Randomized, double‐blind clinical trial	N = 30; 83% men; mean age 56 years; mean H&Y: 1.6	Eszopiclone (EZP; 3 mg if age < 65; 2 mg if age ≥ 65) vs. PLB	6 weeks	Diary, 10‐point sleep quality scale, PDQ‐8, CGI‐I (sleep), FSS, daytime alertness	Diary showed fewer awakenings with EZP vs. PLB (1.0 vs. 1.8*); CGI‐I for sleep improved more with EZP vs. PLB (2.3 vs. 3.2*); sleep quality improved with EZP vs. PLB (5.0 vs. 5.3*).
Avila et al, 2015^60^	Prospective observational cohort	N = 24; 50% men; age 75 ± 8 years; median H&Y: 2.0; all with depression	Agomelatine (12.5–50 mg) at bedtime	2‐week titration the 22‐week stable dosing	PDSS, SCOPA‐S, PSG	Relative to baseline, improvements in SCOPA night (6.5 vs. 1.0**), PDSS (95 vs. 119***), and PSG awakenings (18 vs. 10*)

* *P* < 0.05;

** *P* < 0.01;

*** *P* < 0.001.

PD, Parkinson's disease; H&Y, Hoehn & Yahr; CBT‐I, Cognitive Behavioral Therapy for Insomnia; BLT, bright light therapy; PDSS, Parkinson's Disease Sleep Scale; PSQI, Pittsburgh Sleep Quality Index; ISI, Insomnia Severity Index; SCOPA‐S, Scales for Outcomes in Parkinson's Disease; PGI‐C, patient global impression of change; CGI‐C, clinical global impression of change; ESS, Epworth sleepiness scale; FSS, Fatigue Severity Scale; PDSS‐2, Parkinson's disease sleep scale 2; PLB, placebo; CDLD, carbidopa–levodopa; CR, continuous release; TST, total sleep time; SOL, sleep onset latency; PR, prolonged release; CI, confidence interval; SR, sustained‐release; IR, immediate‐release; ROT, rotigotine; PPX, pramipexole; NMSS, Non‐Motor Sleep Scale; APO, apomorphine; IJLI, intrajejunal levodopa infusion; AE, adverse events; SSS, Stanford Sleepiness Scale; GSDS, General Sleep Disturbance Scale; NR, not reported; PSG, polysomnography; LS, least squares; PDQ‐8, Parkinson's Disease Questionnaire, Short Form; CGI‐I, clinical global impression of improvement; GSDS, General Sleep Disturbance Scale; EZP, eszopiclone.

In general, the use of long‐acting dopamine agonists has produced modest improvements of insomnia symptoms (Table 2). In the Efficacy and Safety Evaluation in PD RCT, individuals with advanced PD randomized to adjunctive ropinirole prolonged release (PR) showed stabilization of the PDSS relative to placebo for 24 weeks.^48^ In a secondary analysis of the Efficacy and Safety Evaluation in PD RCT, Chaudhuri and colleagues^49^ examined the effects of ropinirole PR versus placebo among individuals with advanced PD by insomnia status. Participants reporting insomnia symptoms (PDSS ≤100) at baseline and randomized to ropinirole PR (n = 93) experienced greater improvements in the PDSS at 12 and 24 weeks relative to placebo (n = 8 9). Mild but significant improvements were observed in the insomnia items (ie, global sleep quality) and motor symptoms upon awakening. A similar pattern of improvement of insomnia symptoms among individuals with advanced PD and insomnia symptoms (PDSS <90) was observed with the addition of pramipexole sustained release or immediate release (IR).^50^ Although individuals experienced significant increases in the PDSS total, global sleep quality, and nocturnal motor symptoms subscales over time with either pramipexole formulation, individuals receiving the sustained release experienced larger (but nonsignificant) improvements over the IR.

The rotigotine patch may also benefit sleep symptoms in advanced PD. However, the sleep benefits of rotigotine may be comparable to that of shorter acting dopamine agonists. For example, in the Clinical Efficacy of Pramipexole and Transdermal Rotigotine in Advanced PD trial, 505 individuals with PD and motor fluctuations were randomized to rotigotine, pramipexole IR (3 times daily), or placebo.^51^ Both rotigotine and pramipexole were significantly better than placebo at improving the PDSS total scores (Table 2). The magnitude of the PDSS improvement was similar between these 2 medications (difference 0.6; *P* = 0.20 rotigotine vs. pramipexole IR). In another RCT, individuals with PD and motor fluctuations were randomized to treatment with rotigotine, ropinirole IR (3 times daily), or placebo.^52^ Both rotigotine and ropinirole were significantly better than placebo at improving the PDSS‐2 scores. The improvement on the PDSS‐2 was statistically equivalent between these 2 agents (difference − 0.7; *P* = 0.28 rotigotine vs. ropinirole). In a study of 287 PD patients with early‐morning motor symptoms, rotigotine patch (dosed up to 16 mg/24 hours) was significantly better than placebo at improving scores on the PDSS‐2 and its subscales (including “disturbed sleep”). The NMS scale also was significantly improved with rotigotine, including the sleep/fatigue subscore.^53^ Because of the dosing convenience over shorter acting dopamine agonists, the use of the rotigotine patch may be preferred among individuals with PD experiencing motor fluctuations and insomnia.

Observational studies of alternative therapies for providing consistent dopaminergic stimulation have produced positive effects on insomnia symptoms in advanced PD with motor fluctuations. For example, a prospective study comparing individuals receiving subcutaneous apomorphine or intrajejunal levodopa gel infusion showed that both treatments significantly improved the NMS sleep/fatigue domain.^54^ However, a larger treatment effect for sleep improvement was seen for those treated with intrajejunal levodopa gel infusion (Cohen's d = 0.7) than for apomorphine infusion (Cohen's d = 0.4). Another open‐label study examining 38 individuals treated with intrajejunal levodopa gel infusion also significantly improved most NMS scale domains.^55^ Improvements from baseline in the NMS scale sleep/fatigue domain were noted at 12 weeks (52% reduction, *P* < 0.001) and 60 weeks of therapy (47% reduction, *P* < 0.001). Thus, these data support the hypothesis that medications that provide stable plasma levels of dopamine may improve sleep fragmentation in PD.

### Nondopaminergic Medications for Insomnia in PD

The following 3 sedative–hypnotic treatments have been tested for insomnia in PD patients, with mixed results: eszopiclone, doxepin, and melatonin (Table 2). Eszopiclone is Food and Drug Administration–approved for the treatment of DIS and DMS. In a single RCT including 30 PD patients, a dose of 2 to 3 mg (depending on patient age) significantly improved several sleep measures when compared with placebo.^56^ These included a reduction in nocturnal awakenings and improvement in sleep quality ratings. Eszopiclone side effects occurred in 13% of the treatment group and included daytime sleepiness and dizziness.

Doxepin is also Food and Drug Administration–approved for the treatment of DIS and DMS at doses of 3 and 6 mg. In a 3‐arm parallel trial of 18 participants, doxepin 10 mg was compared to red light (placebo condition) and the combination of cognitive behavioral therapy for insomnia (CBT‐I) and bright light therapy (BLT). Compared with the placebo condition, the doxepin group had significant improvements in ISI scores, SCOPA‐S, and fatigue.^57^ Of the participants in the doxepin group, 50% reported side effects, including fatigue, orthostatic dizziness, and nausea.

Melatonin is not a Food and Drug Administration–approved medication, but it is sometimes recommended for insomnia. Two RCTs have evaluated melatonin for the treatment of insomnia in PD. In the first, 40 participants completed a crossover study with the following 3 arms: melatonin 5 mg, melatonin 50 mg, and placebo.^58^ Significant improvements were reported for 5 mg melatonin versus placebo on the General Sleep Disturbance Scale, and significant improvements were reported for 50 mg melatonin on total sleep time. Other measures, including the PSQI, were unchanged by either melatonin dose. In the second study, 18 people with PD were randomized to melatonin 3 mg or placebo. In contrast to the prior study, in this study melatonin significantly improved PSQI scores when compared with placebo, whereas PSG measures of sleep were unchanged.^59^

Two studies have examined treating mental health comorbidities in PD and its impact on insomnia symptoms. In a prospective cohort, 24 individuals with PD and depression were treated with agomelatine, a melatonin MT1/MT2 receptor agonist and serotonin 5HT_2c_ receptor antagonist (available in Europe and Australia).^60^ Relative to baseline, significant improvements were reported for depressive symptoms, PDSS, SCOPA‐S, and polysomnographic number of awakenings (Table 2). In 2 RCTs, individuals with PD and psychosis were treated with pimavanserin, a serotonin 5HT_2A_ receptor inverse agonist/antagonist.^61^ In a pooled analysis of these trials, relative to placebo (n = 67), individuals with baseline insomnia symptoms (n = 69; SCOPA‐night ≥7) treated with pimavanserin 34 mg showed greater improvement in their nighttime complaints (SCOPA‐night −4.4 vs. −2.6, *P* = 0.002). Although these studies did not have insomnia as a primary endpoint, these investigational data show that for some individuals with PD psychiatric nonmotor symptoms may covary with insomnia.

### Movement Disorder Society's Evidence‐Based Medicine Review for the Treatment of Nonmotor Symptoms in PD

As part of a comprehensive nonmotor symptom treatment review, the Movement Disorder Society examined the literature for the efficacy, safety, and practice implications of insomnia treatments in PD.^62^, ^63^ The medications that were considered “possibly useful” in clinical practice with acceptable risk safety profiles were rotigotine, eszopiclone, and low‐dose melatonin 3 to 5 mg. The data for the use of carbidopa–levodopa CR and high doses of melatonin (50 mg) to treat insomnia were judged insufficient in terms of efficacy and considered “investigational” for clinical use.

### Pharmacologic Treatment of Insomnia in Non‐PD Populations: Evidence‐Based Recommendations

Given the few studies of pharmacologic treatment of insomnia of individuals with PD, it is difficult to conclusively recommend any particular medication treatment strategy. However, many more studies of insomnia treatment have been performed in non‐PD populations. Recently, the American Academy of Sleep Medicine (AASM) published clinical practice guidelines with evidence‐based recommendations for pharmacologic treatment of insomnia.^64^ For DIS, the AASM gave conditional recommendations in favor of the z‐drugs (zaleplon, zolpidem, eszopiclone), ramelteon, temazepam, and triazolam. For DMS, it gave conditional recommendations in favor of zolpidem, eszopiclone, doxepin, temazepam, and suvorexant. The AASM guideline suggested that clinicians not use the commonly recommended agents trazodone or diphenhydramine because of a lack of meaningful treatment benefit in available studies.

### Risk of Pharmacologic Treatment of Insomnia in Non‐PD Populations

The American Geriatrics Society maintains a listing of potentially inappropriate medications for use in older adults (≥ 65 years old), known as the Beers criteria.^65^ Although not all patients with PD are geriatric, and side effects in people with PD will not necessarily exactly mirror those of geriatric patients in general, these criteria provide an assessment of medications with potential for harm (eg, falls) in a vulnerable population. The Beers criteria were updated in 2019.^65^ Of the nondopaminergic medications studied for insomnia in PD patients (ie, eszopiclone 2–3 mg, doxepin 10 mg, and melatonin), these new criteria give a strong recommendation against use of all z‐drugs, including eszopiclone, and against the use of doxepin at doses >6 mg. No recommendation against melatonin was given. The broader insomnia treatment recommendations from the AASM also include benzodiazepines, ramelteon, and suvorexant.^64^ Of these, the Beers criteria give a strong recommendation against use of benzodiazepines, although with the caveat that benzodiazepines may be appropriate for some indications such as RBD.^65^ There are few safety data concerning suvorexant use in PD. Therefore, in the elderly patient with PD and insomnia, preferential consideration may be given to low‐dose melatonin formulations, doxepin <6 mg, or ramelteon 8 mg, although PD‐specific data are lacking for the latter 2 treatments.

## Nonpharmacological Treatments of Insomnia in PD

### Behavioral Interventions

CBT‐I is the first‐line therapy for insomnia as endorsed by the American College of Physicians.^66^ This multicomponent therapy uses sleep restriction and stimulus control to eliminate conditioned hyperarousal as well as cognitive therapy to challenge the dysfunctional sleep beliefs that perpetuate insomnia.^24^ Despite its wide acceptance as the preferred therapy for insomnia, only 2 small RCTs have examined CBT‐I in those with PD (Table 3). In 1 study, Rios‐Romenets and colleagues^57^ compared 6 individuals receiving CBT‐I combined with BLT to 6 control subjects for 6 weeks. Individuals receiving CBT‐I/BLT experienced a significant reduction in ISI scores when compared with controls; however, conclusions about CBT‐I alone cannot be made because of the dual treatment tested. In a second study, Patel and colleagues^67^ examined the delivery of interactive, on‐line CBT‐I to a sleep hygiene education control. Individuals completing the CBT‐I intervention had a greater reduction in the ISI when compared with controls (−7.9 vs. −3.5; *P* = 0.03). However, the drop‐out rate for the CBT‐I group was 43%. Neither of these RCTs reported significant major side effects related to CBT‐I. Thus, these data suggest that CBT‐I is a safe and efficacious treatment, but strategies to increase convenience and enhance treatment adherence in PD are needed.

**Table 3:** *Studies examining nonpharmacologic interventions for insomnia in PD*

Study	Design	Demographic + PD Staging	Intervention	Treatment Duration	Insomnia/Sleep Assessments	Main Findings
Rios Romenets et al., 2013^57^	Randomized clinical trial	N = 18, 78% men; age 66 ± 12 years; H&Y: 1–3	CBT‐I + daily BLT (n = 6) vs. doxepin 10 mg (n = 6) vs. placebo (red light; n = 6)	6 weeks	PDSS, PSQI, ISI, SCOPA‐S, sleep diary	CBT‐I/BLT significantly reduced the ISI relative to placebo (−7.8 ± 3.8 vs. −2.0 ± 3.9; *P* = 0.03). No other significant improvements in sleep noted
Patel et al., 2017^67^	Randomized clinical trial	N = 28, 57% men; age 64 ± 8 years; H&Y: NR	Online CBT‐I (n = 14) vs. sleep hygiene control group (n = 14)	6 weeks	ISI	Among completers, ISI reduction was significantly better in CBT‐I than the control group (ISI: −7.9 vs. −3.5, *P* = 0.03)
Videnovic et al., 2017^68^	Randomized clinical trial	N = 31, 42% men; age 62 ± 10 years; H&Y: 2.0 ± 0.4	1‐hour BLT (10 K lux; n = 16) or dim light (placebo; n = 15) in am and evening	2 weeks	Sleep diaries, PSQI, PDSS	Group‐by‐time interactions noted with the BLT group improving more in SD DIS and number of awakenings, PSQI, and PDSS than the placebo group
Martino et al., 2018^69^	Retrospective longitudinal	N = 140, 65% men; age 66 ± 10 years; H&Y: NR	Daily 1‐hour BLT (3–4K lux) 1 hour before usual bedtime	4 months–15 years	Investigator‐derived insomnia Likert scale: B, 1, 2, 4, 6 minutes and Q 6 minutes	Main effects for insomnia improvement with BLT were noted during the 5‐year period. Rapid improvement occurred in the first month of BLT, continued to improve in year 1, and then plateaued
Nascimento et al., 2014^73^	Prospective cohort	N = 42, 50% men; age 67 years; H&Y: 1.7	Multimodal 1‐hour exercise sessions 3x/wk (n = 23) vs. control group (n = 19)	6 months	MSQ	Group‐by‐ time interaction noted with exercise group modestly improving on MSQ while control group worsened
Frazzitta et al., 2015^70^	Retrospective	N = 138, 44% men; age 69 ± 7 years; H&Y: 2.6 ± 0.5	Multidisciplinary exercise 3 1‐hour sessions/d 5x/wk (n = 89) vs. control group (n = 49)	28 days	PDSS	Group‐by‐time interaction noted with PDSS scores improving in exercise group (107 ± 27 to 118 ± 20; *P* < 0.001) while they slightly worsened in the control group (113.3 ± 14.8 vs. 112.8 ± 14.8; *P* = 0.03)
Silva‐Batista et al., 2017^71^	Randomized clinical trial	N = 22, 73% men; age 65 ± 9 years; H&Y: 2.5 ± 0.5	Resistance training 1‐hour sessions 2x/wk (n = 11) vs. control group (n = 11)	12 weeks	PSQI	Group‐by‐time interaction noted with PSQI sleep quality, sleep disturbance, and daytime dysfunction scores improving in resistance training group while no changes were observed in the control group. No improvements noted in SOL, sleep duration, or sleep efficiency
Xiao and Zhuang, 2016^75^	Randomized clinical trial	N = 96, 70% men; age 68 ± 9 years; H&Y: 2.2 ± 0.2	Qigong minimum of 4x/wk + 30‐minute walking/d (n = 48) vs. control condition (30‐minute walking/d; n = 48)	6 months	PDSS‐2	Group‐by‐time interactions noted with Qigong group improving in PDSS‐2 total score (29 ± 13 to 15 ± 11; *P* = 0.04) and all its subscales while no significant changes were noted in controls
Yang et al., 2017^76^	Randomized clinical trial	N = 36, 56% men; age 63 ± 5; H&Y: 1–3	Tai Chi group‐based sessions (n = 19) vs. Tai Chi individual sessions (n = 17); session 3x/wk in both conditions	13 weeks	PDSS	Both Tai Chi groups showed significant improvements of PDSS total score over time (group: 97 ± 16 to 108 ± 16; individual: 97 ± 20 to 105 ± 20; both changes *P* < .001)

PD, Parkinson's disease; H&Y, Hoehn & Yahr; NR, not reported; CBT‐I, Cognitive Behavioral Therapy for Insomnia; BLT, bright light therapy; wk, week; PSQI, Pittsburgh Sleep Quality Index; PDSS, Parkinson's Disease Sleep Scale; ISI, Insomnia Severity Index; MSQ, Mini‐Sleep Questionnaire; SD, sleep diary; DIS, difficulty initiating sleep; SOL, sleep onset latency; PDSS‐2, Parkinson's disease sleep scale 2.

### BLT

A total of 2 studies have used BLT to improve sleep quality or insomnia symptoms in PD (Table 3). In a RCT, Videnovic and colleagues^68^ randomized sleepy individuals to either BLT or dim light (control condition) and examined sleep‐related outcomes. Although insomnia symptoms were not among the inclusion criteria, all individuals enrolled had poor sleep quality (PSQI >5). Each group received 1 hour of light therapy twice daily (morning, early evening) for 14 days. Although both groups improved in sleep metrics, individuals receiving BLT had greater improvements in DIS, PSQI, and PDSS scores than those receiving dim light. In a retrospective longitudinal series of individuals with PD and sleep problems (n = 140), Martino and colleagues^69^ described the effects of 1 hour of daily BLT before bedtime on insomnia complaints. Insomnia improved within the first month of BLT, and these improvements increased during the 5‐year study period. Taken together, these data suggest that BLT may be an appropriate option for individuals with more advanced PD unable to engage in other nonpharmacological interventions for insomnia.

### Exercise

Exercise interventions have been found to alleviate insomnia in individuals with early to moderate PD (Table 3). For example, in individuals in a rehabilitation hospital, Frazzitta and colleagues^70^ performed a retrospective analysis of individuals completing a 28‐day, multimodal, intensive exercise regimen (3 hr/d) and comparing them with nonexercising controls. The exercise group had significant improvements in the PDSS sleep quality, DIS, DMS, and restlessness items, whereas the control group showed no change. In a smaller RCT, Silva‐Batista and colleagues^71^ compared a 12‐week progressive resistant training (RT) intervention to a nonexercising education control condition. The RT group performed 5 RT exercises for 1 hour twice a week. Upon completion, the RT group had significant improvements in the PSQI sleep quality and sleep medication subscores. In contrast, sleep improvements were not observed in the control group. Additional studies have also shown that low levels of weekly physical activity are sufficient to alleviate some insomnia symptoms in PD.^72^, ^73^ Although untested in PD, hypothetically, exercise may improve sleep by boosting homeostatic sleep drive and strengthening circadian entrainment. However, individuals must be consistently willing and able to participate.

### Qigong/Tai Chi

Qigong, a mind–body therapy grounded in traditional Chinese medicine, has been used as an alternative/holistic treatment in studies exploring sleep in mild and moderate PD (Table 3). This low‐intensity physical activity uses meditative movements and breathing exercises to achieve relaxed states.^74^ In the largest RCT to date (n = 100), the effects of 1 to 4 weekly sessions of Qigong plus daily walking were compared with walking alone for 6 months.^75^ The Qigong group experienced greater improvements across all PDSS‐2 subscores (including disturbed sleep) than the control group. Tai Chi, a martial arts version of Qigong, has also been shown to improve PDSS sleep quality similarly when delivered in group settings or individually for 13 weeks.^76^ Similar to exercise interventions, Qigong and Tai Chi have been associated with improvement in gait function and several other nonmotor symptoms of PD.^75^, ^76^

### Deep Brain Stimulation

Insomnia symptoms are among the nonmotor symptoms improved by deep brain stimulation (DBS) implantation. Most studies have examined subthalamic nucleus DBS and have shown improvements in PSQI or PDSS scores from pre‐ to postimplantation at variable periods (1 week–26 months).^77^, ^78^ Specifically, significant improvements occurred in most PSQI subscores (ie, sleep quality, sleep latency, sleep efficiency, sleep duration) and PDSS sleep quality, sleep onset, and maintenance insomnia items.^77^, ^78^ In addition, improvements in objective PSG parameters have also been noted (wake after sleep onset time, sleep efficiency). Although insomnia is not a primary indication for DBS, improvements in sleep may be an added benefit. Because DBS is typically performed as a treatment for medically refractory motor fluctuations, it may be that the insomnia benefit is because of the amelioration of these symptoms. Whether DBS would be helpful for insomnia in other PD populations is not known.

## Insomnia Treatment Recommendations in PD

**Figure 2:**
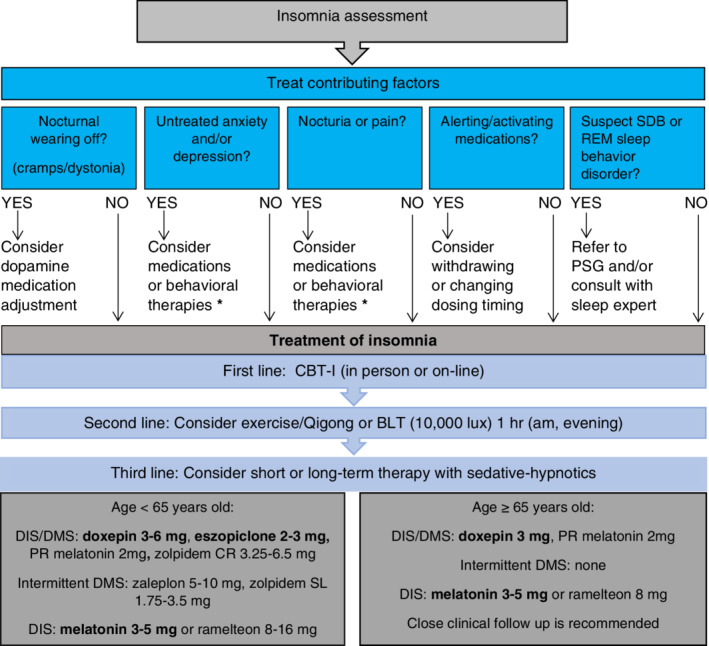
Insomnia treatment algorithm. Bold medications represent PD‐specific data. BLT, bright light therapy; CBT‐I, cognitive behavioral therapy for insomnia; CR, continuous release; DIS, difficulty initiating sleep; DMS, difficulty maintaining sleep; PD, Parkinson's disease; PR, prolonged release; PSG, polysomnography; REM, rapid eye movement; SDB, sleep disordered breathing; SL, sublingual. *Consider “clinically useful treatments” as per the Movement Disorder Society's Evidence‐Based Medicine Reviews of Treatment for Nonmotor Symptoms of Parkinson's disease.^62^, ^63^

We present an algorithm of insomnia treatment recommendations synthesizing available PD and general literature and experience from our clinical practice (Fig. 2).^62^, ^63^, ^64^, ^65^ First, contributory comorbid conditions (eg, sleep disorders, nocturnal motor and nonmotor symptoms) should be identified and addressed. The treatment of comorbid sleep disorders (eg, SDB, RBD) may require collaboration with a sleep specialist, whereas the treatment of comorbid psychiatric symptoms may require collaboration with a psychiatrist or therapist.

In some individuals, these comorbid conditions may be the cause of the insomnia, whereas in others they may merely be correlated. Upon initial assessment, it may be unclear whether the comorbid condition is causing the insomnia or spuriously correlated. Adequate management of comorbid conditions may be sufficient to resolve insomnia in some cases. However, in other cases, insomnia remains a residual symptom after the primary condition remits. Thus, the relationship between a comorbid condition and insomnia may/should be disentangled during follow‐up visits and addressed in individuals where it persists.

After potential contributing factors have been considered (Fig. 2), behavioral interventions (eg, CBT‐I) can be considered potential first‐line therapy. Although a review of sleep hygiene is worthwhile in all individuals with sleep difficulties (eg, avoiding caffeine/alcohol, napping), improving sleep practices alone rarely results in clinically significant improvements in insomnia.^79^ Thus, most individuals interested in behavioral therapies would benefit from CBT‐I. Local CBT‐I therapy providers may be identified by searching the Society for Behavioral Sleep Medicine website or asking local sleep centers. Identifying providers familiar with PD is important as CBT‐I instructions may need modifications such as allowing short daytime naps. To alleviate burden, self‐guided online versions of CBT‐I are also available on public platforms (eg, http://www.veterantraining.va.gov/insomnia). Exercise and Qigong may be considered next, provided physical/cognitive impairments do not preclude it. For individuals wanting to try other nonpharmacologic options, BLT may also be considered. As per the protocol of Videnovic and colleagues,^68^ we recommend using BLT 10,000 lux for 1 hour twice daily (morning, evening). Although there are few data for BLT in insomnia and PD because it is safe and carries a low patient burden, it may be a viable option for many individuals.

In general, pharmacotherapy for insomnia should be considered second‐line or third‐line therapy. However, there may be individuals who prefer pharmacotherapy for insomnia first or in whom it may be most appropriate because of limitations in access or ability to engage in nonpharmacologic approaches. We suggest pharmacotherapeutic options based on the Beers criteria, AASM guidelines, and the Movement Disorder Society's treatment of nonmotor symptoms evidence‐based reviews.^62^, ^63^, ^64^, ^65^ For individuals with chronic DIS and DMS, the general approach is to select an agent with a longer half‐life to keep the individual asleep most of the night. In younger individuals (<65 years old), the choices include PR melatonin, low‐dose doxepin (3–6 mg), or eszopiclone (2–3 mg). Although data in PD are lacking, CR zolpidem (6.25–12.5 mg) may be another option for DIS/DMS. For individuals who intermittently have DMS alone (middle of the night awakenings), the choices could include zaleplon 5 to 10 mg or zolpidem sublingual tablets (1.75–3.5 mg), which both have short half‐lives. For individuals with isolated DIS, low‐dose melatonin 3–5 mg or ramelteon 8–16 mg may be considered.

For older individuals (≥65 years old), the lowest doses of any of these agents may be considered, but low‐dose (3–5 mg) IR or PR melatonin, doxepin <6 mg, or ramelteon 8 mg might be preferentially chosen based on the Beers criteria recommendations against the other agents. However, even these carry the risk of significant side effects.^57^ Thus, close clinical follow‐up and caregiver supervision of medication administration are important. Given the risk of not only physical but also psychological dependence on sedative–hypnotics, we generally advise against long‐term use in most cases and note that there are few safety data concerning the long‐term use of sedative–hypnotic medications in PD. Nonetheless, we recognize that insomnia is often a chronic problem.

## Limitations

This review article has several limitations. First, our search criteria may have not captured all studies of insomnia symptoms in PD. In addition, many studies reported sleep symptoms as aggregated scores (ie, total PDSS as opposed to its subscales or individual items). These studies were excluded because specific insomnia symptoms could not be identified or treatment effects assessed. Finally, a limitation of our proposed algorithm is that many of these pharmacological agents may not be available outside of the United States.

## Conclusion and Future Directions

The purpose of this clinical review is to provide guidance on the pragmatic assessments and treatments of insomnia in PD. However, there are many unresolved issues. Given the relative paucity of data, robust clinical trials for insomnia in PD are needed. The safety and efficacy of new (ie, suvorexant) and existing agents and behavioral interventions need to be evaluated in older and younger individuals throughout the spectrum of PD. By drawing attention to the gaps in the literature, we hope to stimulate needed research about this prevalent sleep disorder in PD.

## Author Roles

(1) Research Project: A. Conception, B. Organization, C. Execution; (2) Manuscript Preparation: A. Writing of the First Draft, B. Review and Critique.

R.A.H.: 1A, 1B, 1C, 2A

D.M.W.: 1A, 1C, 2A

W.K.W.: 1A, 1C, 2A

L.M.T.: 1A, 1C, 2A

I.A.M.: 1A, 2B

S.A.F.: 1A, 2B

A.W.A.: 1A, 2B

L.W.: 1A, 2B

S.N.: 1A, 2B

## Disclosures

**Ethical Compliance Statement:** Given this manuscript is a review article, institutional review board approval was unnecessary. Informed patient consent was not necessary for this work. We confirm that we have read the Journal's position on issues involved in ethical publication and affirm that this work is consistent with those guidelines.

**Funding Sources and Conflicts of Interest:** This work was supported by an educational grant from Neuro Challenge Foundation, a Parkinson's disease advocacy group from Sarasota, FL.

**Financial Disclosures for Previous 12 Months:** Dr. Robert Hauser has the following disclosures: he reports consulting fees from Acadia Pharmaceuticals, Acorda Therapeutics, Adamas Pharmaceuticals, AlphaSights, Amneal Pharmaceuticals, Inc., ApoPharma Inc., Aptis Partners LLC, CNS Ratings LLC, Compass Group, Decision Resource Group, Dedham Group, Defined Health, Enterin Inc., Extera Partners LLC., Gerson Lehman Group Inc., Global Kinetics Corporation, Guidepoint Global, Health Advances, Impax Lab, Impel Neuropharma, IntraMed Educational Group, IQVIA, International Stem Cell Corporation, Jazz Pharmaceuticals, Kashiv Pharma, L.E.K Consulting, Lundbeck A/S, Lundbeck LLC, Med IQ, Medscape, Mitsubishi Tanabe Pharma America, Michael J Fox Foundation, Neuro Challenge Foundation for Parkinson's, Neurocrea LLC, Neurocrine Biosciences Inc, Neurocrine Continental, Inc., Northwestern University, Orbees Inc., Orion, Parkinson's Foundation, Partners Healthcare, Penn Technology Partnership, Perception OpCo, Prescott Medical Communications Group, Prilenia Therapeutics LLC, Parkinson's Study Group, Regenera Pharma, Scion Neurostim LLC, Seelos Therapeutics, Slingshot Insights, Sunovion Pharmaceuticals Inc., Teva Pharmaceuticals, US World Meds, and WebMD. Dr. Hauser reports research support from AbbVie Inc., Acorda Therapeutics, AstraZeneca, Axovant Sciences, Biogen Inc., Cavion, Enterin Inc., Impax Laboratories, LLC., Intec Pharma Ltd, Jazz Pharmaceuticals, NeuroDerm Ltd., Lundbeck, Michael J Fox Foundation for Parkinson's Research, F. Hoffman‐La Roche, Dart NeuroScience LLC, Prexton Therapeutics, Revance Therapeutics Inc., and Sunovion Pharmaceuticals. Dr. Malaty has the following disclosures: she participated in research funded by the Parkinson Foundation, Tourette Association, Dystonia Coalition, Abbvie, Auspex, Biogen, Biotie, Intrepid, Lily, Lundbeck, Neurocrine, Pfizer, Revance, and Teva, but has no owner interest in any pharmaceutical company. She has given educational talks for Medscape, the Cleveland Clinic, the Tourette Association of America, the NeuroChallenge foundation, and NIH/Neurobiology of Disease in Children conference with either compensated travel or honorarium and has coauthored a Parkinson's disease book for Robert Rose publishers with royalties. Dr. Factor has the following disclosures: he has received honoraria from Lundbeck, Teva, Sunovion, Biogen, Acadia, and Neuroderm. He has participated in research funded by Ipsen, Medtronics, Boston Scientific, Teva, US World Meds, Sunovion Therapeutics, Vaccinex, Voyager, Jazz Pharmaceuticals, CHDI Foundation, Michael J. Fox Foundation, and the NIH. He receives royalties from Demos, Blackwell Futura for textbooks, and Uptodate. Dr. Amara has the following disclosures: she is a consultant for Grey Matter Technologies and Jazz Pharmaceuticals. She serves as site principal investigator for studies sponsored by Michael J. Fox Foundation for Parkinson's Research, Biogen Idec, Hoffman La Roche, Eli Lilly, Axovant Sciences, Ltd., and AbbVie Laboratories, all unrelated to this project. Dr. Wallace has the following disclosures: he reports consulting fees from Gerson Lehman Group Inc., Neuro Challenge Foundation for Parkinson's, and L.E.K. Consulting. Drs. Wohlgemuth, Trotti, Wittine, and Nallu have no conflicts of interest relevant to this work.

## Supporting information


**Appendix S1:** Supplementary Material

**Supplementary Figure S1:** search flow diagram

**Supplementary Table S1:** Tools to assess insomnia in Parkinson's disease
