# Supplementary material for: Practical Evaluation and Management of Insomnia in Parkinson's Disease: A Review
Source: Mov Disord Clin Pract. 2020 Feb 3;7(3):250–66. doi: 10.1002/mdc3.12899 (PMC7111581; doi:10.1002/mdc3.12899)
Supplement: Supplementary file 1 — Appendix S1: Supplementary Material [file MDC3-7-250-s001.docx]

*Supplementary Material 1*

*Search strategy details*

The search was conducted in PubMed (Medline), Embase, and Web of Science Plus. Key search terms included “insomnia” or “sleep quality” and “Parkinson’s disease”. The search date spanned January 1998 to December 2018. Articles that met the criteria for the review included those that: 1) included individuals with Parkinson’s disease (PD), 2) were primary studies, 3) were published in peer-reviewed journals, 4) were written in English and 5) assessed/defined insomnia. Two authors (DMW, WKW) independently reviewed the titles followed by the abstracts of each article. Articles meeting the inclusion criteria were fully reviewed by the authors to extract additional details. Although we primarily included randomized, clinical trials for the treatment of insomnia, we also included observational studies in areas (i.e. behavioral therapies) where data were sparse. Given the educational aspect of this review and reference limitations, we selected the highest quality representative interventional studies. To capture most relevant studies on this topic, we also conducted an ancestry search of retrieved articles’ reference lists.

*Supplementary Material 2*

*Non-exclusive insomnia assessment instruments*

Several instruments exist which include insomnia as one component of a broader assessment.

One of the most commonly used for this purpose is the Pittsburgh Sleep Quality Index (PSQI) ^9^. It consists of 19 items and uses 7 sub-scores to assess an individual’s sleep over the previous month ^9^. It queries total sleep time (TST), sleep onset latency (SOL), sleep efficiency (TST/ time in bed), sleep disturbances (e.g. sleep disordered breathing [SDB] risk), use of sleep medications, and daytime function. Global PSQI scores > 5 indicate poor sleep quality but it does not determine its etiology ^9^. However, nothing in the PSQI is specific to PD.

Two scales have been developed and used to specifically assess sleep difficulty in PD: 1) Scales for Outcomes in Parkinson’s Disease-Sleep (SCOPA-S) and 2) Parkinson’s Disease Sleep Scale (PDSS). The SCOPA-S assesses an individual’s sleep over the last month across four domains: 1) sleeping pill use, 2) insomnia complaints, 3) sleep quality, and 4) daytime sleepiness ^10^. Specifically, the frequency of difficulty initiating sleep (DIS), difficulty maintaining sleep (DMS), early morning awakenings (EMA), and multiple awakenings are assessed. The PDSS evaluates not only insomnia, but a wide spectrum of nocturnal PD symptoms which occurred during the previous week. The original PDSS consisted of 15 items, with lower scores indicating poorer sleep quality ^11^. The first three items assess 1) global sleep quality, 2) DIS, and 3) DMS. Other nocturnal symptoms queried include restlessness, nocturia, dystonia, hallucinations, and vivid dreams. Importantly, the PDSS queries whether these factors contribute to insomnia. A revision of this scale, the PDSS-2, changed the method of response to improve ease of completion ^12^. In addition, the PDSS-2 added new questions about other sleep disorders (i.e. SDB, restless legs syndrome). The PDSS-2 covers three domains: nocturnal motor symptoms, PD-specific nocturnal symptoms, and disturbed sleep. In the PDSS-2, unlike the original PDSS, higher scores indicate greater sleep impairments. A score ≥ 15 discriminates poor from good sleepers with a sensitivity of 72% and specificity of 73% ^13^ .
