# Supplementary material for: Practical Evaluation and Management of Insomnia in Parkinson's Disease: A Review
Source: Mov Disord Clin Pract. 2020 Feb 3;7(3):250–66. doi: 10.1002/mdc3.12899 (PMC7111581; doi:10.1002/mdc3.12899)
Supplement: Supplementary file 2 — Supplementary Figure S1: search flow diagram [file MDC3-7-250-s002.docx]

Supplementary Figure: search flow diagram

611 records identified

209 records excluded: not about insomnia or Parkinson’s disease

98 studies included in the analysis

304 full text articles excluded:

170 reviews or meta-analyses

64 studies with participants < 15

70 studies failed to provide insomnia assessment /definition

402 full text articles reviewed for eligibility

611 records screened

575 records identified through database search

36 additional articles identified through other sources (ancestry)
