# Supplementary material for: Practical Evaluation and Management of Insomnia in Parkinson's Disease: A Review
Source: Mov Disord Clin Pract. 2020 Feb 3;7(3):250–66. doi: 10.1002/mdc3.12899 (PMC7111581; doi:10.1002/mdc3.12899)
Supplement: Supplementary file 3 — Supplementary Table S1: Tools to assess insomnia in Parkinson's disease [file MDC3-7-250-s003.docx]

**Supplementary Table: Tools to assess insomnia in Parkinson’s disease**

| Assessment | Advantages | Disadvantages | Duration |
| --- | --- | --- | --- |
| *Initial evaluation*  **Primary**  Clinical interview  Pittsburgh Sleep Quality Index  Insomnia severity index  PDSS-2  SCOPA-S  **Secondary**  video-PSG  Sleep diaries | -explores insomnia etiology  -excludes insomnia mimics (i.e. sleep disorders)  -assesses sleep aid use  -may identify poor sleep hygiene (behavioral targets)  -explores of multiple sleep dimensions (duration, quality, sleep aid use)  -assesses DIS, DMS, EMA, and daytime dysfunction  -some etiologies of insomnia queried (nocturia, pain)  -strong psychometric properties  -available in many languages  -short completion time  -assesses DIS, DMS, EMA and daytime dysfunction  -strong psychometric properties  -available in many languages  -queries sleep quality, DIS,  and DMS  -specific to/validated in PD  -identifies PD factors and comorbid sleep disorders (i.e. RLS) contributing to insomnia  -available in many languages  -queries sleep quality, DIS, DMS, EMA, and daytime sleepiness  -identifies sleep aid use  -specific to/validated in PD  -available in many languages  -detects latent comorbid sleep disorders (i.e. SDB, RBD)  -provides prospective sleep data over longer periods  -monitors changes in sleep with clinical interventions  -easily available | -sleep experience required  -lengthy completion time  -1 month retrospective  -not specific/validated in PD  -etiology of insomnia unspecified  -elevated scores can result from other sleep disorders  -2 weeks retrospective  -not specific/validated in PD  -etiology of insomnia unspecified  -elevated scores can result from other sleep disorders  -does not identify sleep aid use  -1 week retrospective  -does not query about EMA  -does not assess insufficient sleep time or opportunity  -does not identify sleep aid use  -1 month retrospective  -does not assess sleep opportunity or duration  -does not identify comorbid sleep disorders  -costly  -inconvenient/uncomfortable  -not representative of sleeping at home  -limited use among individuals with cognitive impairment  -daily entries can be burdensome/distressing | 15-25 mins  10-15 mins  5 mins  10-15 mins  5-10 mins  1 night  A few mins each morning over 1-2 wks |
